# Superior Mesenteric Vein Thrombosis in the Setting of Ileitis: Case Report and Literature Review

**DOI:** 10.7759/cureus.11107

**Published:** 2020-10-23

**Authors:** Mohamed Elnaggar, Jagmohan S Jandu, Bryce D Beutler, Sally Leong, Robert McCain

**Affiliations:** 1 Internal Medicine, University of Nevada, Reno School of Medicine, Reno, USA; 2 Internal Medicine, Veterans Affairs Sierra Nevada Health Care System, Reno, USA

**Keywords:** acute mesenteric vein thrombosis, ileitis, superior mesenteric vein thrombosis, thrombosis, intraoperative/postoperative anticoagulation

## Abstract

Acute mesenteric vein thrombosis represents a rare but potentially lethal thrombotic event. Its treatment involves prompt and aggressive anticoagulation therapy. In the perioperative setting, management of the underlying thrombus must be weighed carefully against the risk of bleeding. We describe a 57-year-old man who presented with abdominal pain and was found to have terminal ileitis with concomitant superior mesenteric vein thrombosis.

## Introduction

Acute mesenteric vein thrombosis (AMVT) represents a rare, life-threatening cause of intestinal ischemia. Individuals with underlying cancer or hereditary thrombophilias are most commonly affected. However, idiopathic AMVT has also been described [1]. Patients typically present with abdominal pain, nausea, vomiting, and/or melena [2]. A definitive diagnosis can be established with computed tomography (CT) scanning, which shows a filling defect in the mesenteric vein [3]. Bedside Doppler ultrasound or magnetic resonance venography may also be used for patients in whom iodinated contrast material is contraindicated.

Unfractionated or low-molecular-weight heparin represents the mainstay of management for AMVT. The mortality rate for AMVT approaches 50%; therefore, anticoagulation therapy should be administered even in the presence of active bleeding [4,5]. We describe a 57-year-old man who presented with abdominal pain and was found to have ileitis with concomitant superior mesenteric vein thrombosis. In addition, we discuss the management of thrombosis in the perioperative setting.

## Case presentation

**Figure 1:**
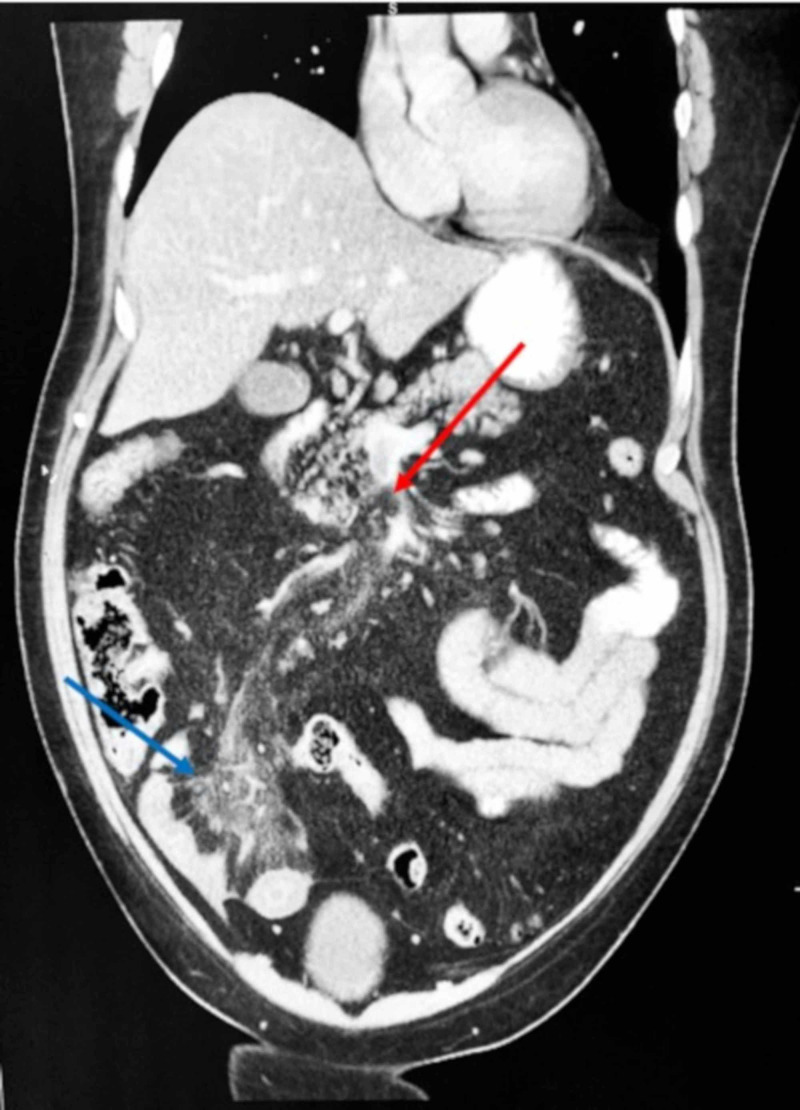
Computed tomography scan of the abdomen and pelvis with intravenous contrast, coronal view, in a 57-year-old man who presented with a one-day history of abdominal pain, nausea, and vomiting. Coronal view of a computed tomography scan of the abdomen and pelvis with intravenous contrast demonstrates a filling defect in the superior mesenteric vein (red arrow) as well as edematous changes in the distal ileum (blue arrow). A diagnosis of superior mesenteric vein thrombosis and concomitant ileitis was established.

A 57-year-old male with a history of dyslipidemia, diabetes mellitus, obesity, and obstructive sleep apnea presented with a one-day history of abdominal pain, nausea, and vomiting. Physical examination revealed exquisite tenderness to palpation of the right lower quadrant. Complete blood count, complete metabolic panel, lipase, and urinalysis were normal. A CT scan of the abdomen and pelvis with intravenous contrast revealed edematous changes in the distal ileum as well as a filling defect in the superior mesenteric vein (SMV) (Figure 1). The patient was started on intravenous antibiotics for suspected ileitis as well as unfractionated heparin for management of SMV thrombosis.

On hospital day three, a repeat CT scan was obtained which demonstrated thrombus regression. The ileitis was unchanged as compared to the previous CT scan. Heparin was held in preparation for colonoscopic evaluation.

A colonoscopy was performed six hours after anticoagulation had been discontinued. Unexpectedly, the terminal ileum appeared normal. However, a 1.2-cm polyp was identified in the ascending colon; this was resected. The gastroenterology team recommended holding anticoagulation therapy for 48 hours following the polypectomy.

**Figure 2:**
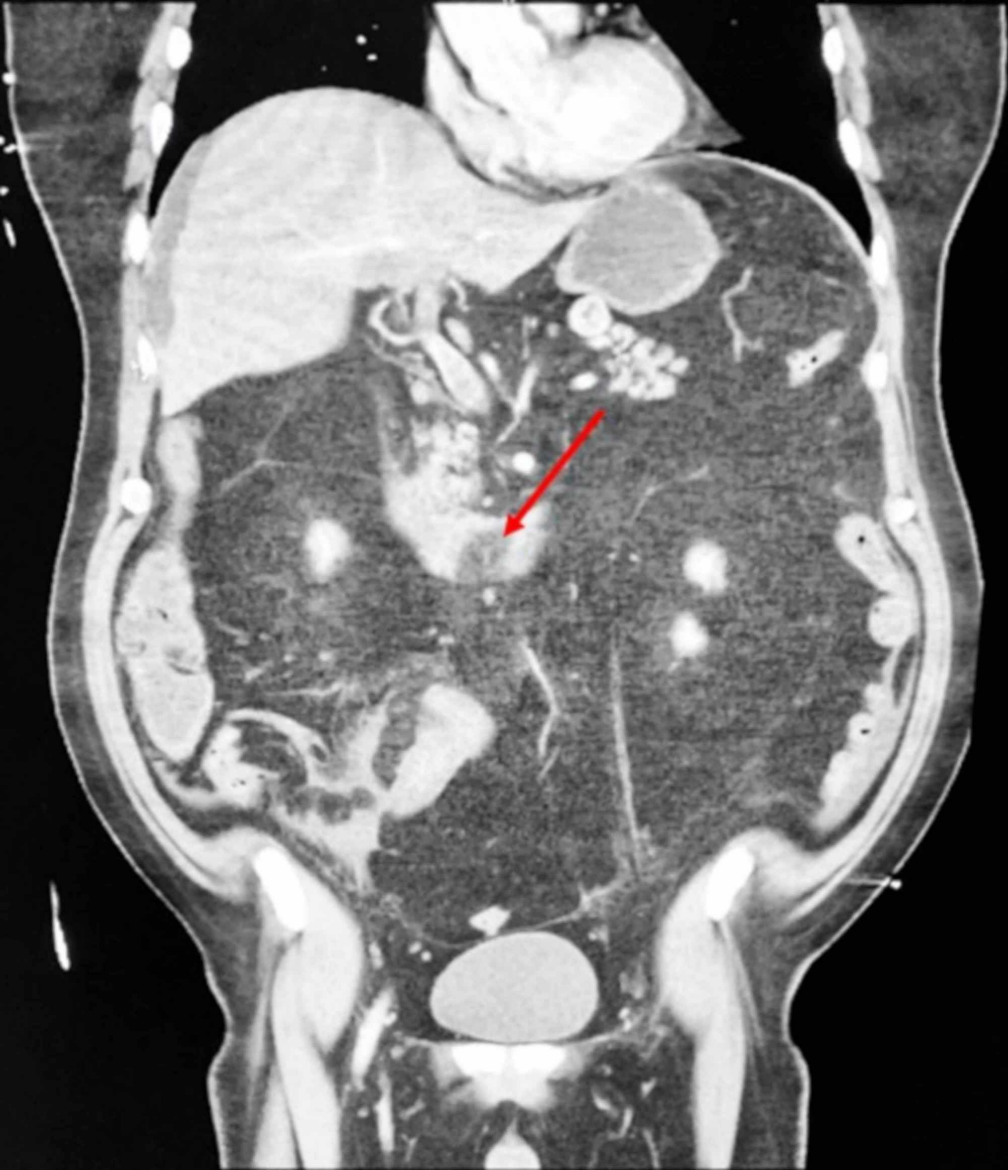
Computed tomography scan of the abdomen and pelvis with intravenous contrast, coronal view, demonstrating a thrombus. Coronal view of a computed tomography scan of the abdomen and pelvis with intravenous contrast revealing a large thrombus in the superior mesenteric vein (red arrow). A computed tomography scan that had been obtained two days earlier had demonstrated thrombus regression; recrudescence likely occurred due to withdrawal of anticoagulation in preparation for a colonoscopy.

The next morning, the patient woke with severe upper abdominal pain. A physical examination revealed marked distention. Laboratory studies revealed leukocytosis (white blood cell count: 20.6 K/uL; reference range: 4.5 - 11.0 K/uL) and lactic acidosis (lactic acid: 7.8 mmol/L; reference range: 0.5 - 2.0 mmol/L). An emergent CT scan was obtained and demonstrated recrudescence of the previously-identified SMV thrombus (Figure 2). An emergent laparotomy revealed extensive small bowel ischemia. As a result, 130 cm of bowel was resected; end-to-end reanastomosis was performed the following day. The pathology of a tissue sample was negative for malignancy.

Anticoagulation therapy with dalteparin was started one day after the reanastomosis. The patient received total parenteral nutrition and was later advanced to a soft diet. He was discharged in stable condition after a three-week hospital stay. Subsequent workup for inherited thrombophilias - including Factor V Leiden, prothrombin gene mutation, and protein C deficiency - was negative.

## Discussion

AMVT classically presents with colicky, periumbilical abdominal pain. Nausea and vomiting are common [6]. Physical examination is often benign, but tenderness to palpation and/or abdominal distention may be present. Fecal occult blood testing is positive in approximately 50% of affected individuals [7]. Other common laboratory findings include leukocytosis and hemoconcentration [5]. Definitive diagnosis can be established via imaging. Magnetic resonance venography represents the gold standard, but CT scan with and without oral and intravenous contrast is faster, more widely available, and approximately 90% sensitive [8]. In addition, some studies have demonstrated sensitivity as high as 99% with Doppler ultrasonography. However, the sensitivity and specificity of this modality are user-dependent, and it does not allow for visualization of the bowel that may be affected in the setting of AMVT [9].

Emergent anticoagulation represents the mainstay of therapy for AMVT [10]. Unfractionated or low-molecular-weight heparin should be administered during the acute phase of thrombosis. Patients may be monitored with serial abdominal examinations, laboratory studies, and/or CT scans to confirm clinical improvement and regression of the thrombus. To the best of our knowledge, there are no clinical practice guidelines pertaining to the interval between repeat CT scans; therefore, clinical evaluation should guide the need for further imaging. Anticoagulation with warfarin or a factor Xa inhibitor should be continued for three to six months thereafter [11]. The risk of major bleeding is less than 10% [12]. Lifelong anticoagulation should be considered for individuals with inherited thrombophilias or other conditions associated with a chronic hypercoagulable state.

The mortality rate for untreated AMVT is approximately 50%; prompt and uninterrupted anticoagulation therapy reduces mortality to nearly 0% [13]. Thus, the value of continuous anticoagulation cannot be overstated. In our patient, a decision was made to interrupt anticoagulation in order to resect a small polyp. The result was recrudescence of the thrombus with dire clinical consequences. This case illustrates the importance of triaging in patients with complicated presentations; indeed, even in the setting of active bleeding, anticoagulation for AMVT should usually be continued [14]. In a scenario in which there are multiple concurrent pathologies, management of the most serious condition should always be prioritized.

An underlying etiology for our patient's AMVT or the imaging appearance of ileitis was never definitively established. We hypothesize that disruption of bowel perfusion may have contributed to edema and thickening of the ileal wall, mimicking an inflammatory process. However, there is no known association between AMVT and the subsequent development of colitis or ileitis.

## Conclusions

AMVT is a rare but potentially life-threatening thrombotic event. Patients typically present with abdominal pain that is out of proportion to physical examination findings. Definitive diagnosis can be established with imaging. Anticoagulation therapy should be administered immediately upon identification of a thrombus and should be continued without interruption for at least three to six months. Indeed, due to the high mortality rate associated with AMVT, anticoagulation should be used even in the presence of active bleeding.
